# Author Correction: Fuzzy recognition by the prokaryotic transcription factor HigA2 from *Vibrio cholerae*

**DOI:** 10.1038/s41467-024-47946-6

**Published:** 2024-04-25

**Authors:** San Hadži, Zala Živič, Matic Kovačič, Uroš Zavrtanik, Sarah Haesaerts, Daniel Charlier, Janez Plavec, Alexander N. Volkov, Jurij Lah, Remy Loris

**Affiliations:** 1 Structural Biology Brussels, Department of Biotechnology, Vrije Universiteit Brussel, Pleinlaan 2, 1050 Brussels, Belgium; 2 Centre for Structural Biology, VIB, Pleinlaan 2, 1050 Brussels, Belgium; 3 Department of Physical Chemistry, Faculty of Chemistry and Chemical Technology, University of Ljubljana, 1000 Ljubljana, Slovenia; 4 Slovenian NMR Center, National Institute of Chemistry, Hajdrihova, 19, 1000 Ljubljana, Slovenia; 5 Research group of Microbiology, Department of Biotechnology, Vrije Universiteit Brussel, Pleinlaan 2, 1050 Brussels, Belgium; 6 Jean Jeener NMR Centre, Vrije Universiteit Brussel, Pleinlaan 2, 1050 Brussels, Belgium

**Keywords:** Intrinsically disordered proteins, Thermodynamics, Structural biology, Transcription factors

Correction to: *Nature Communications* 10.1038/s41467-024-47296-3, published online 10 April 2024

In this article the author name Sarah Haesaerts was incorrectly written as Sarah Haeserts. The original article has been corrected.

