# Supplementary material for: Fuzzy recognition by the prokaryotic transcription factor HigA2 from Vibrio cholerae
Source: Nat Commun. 2024 Apr 10;15:3105. doi: 10.1038/s41467-024-47296-3 (PMC11006873; doi:10.1038/s41467-024-47296-3)
Supplement: Supplementary file 1 — Supplementary Information [file 41467_2024_47296_MOESM1_ESM.pdf]

Supplementary Information

Fuzzy recognition by the prokaryotic transcription factor HigA2 from *Vibrio cholerae*

San Hadži[1, 2, 3], Zala Živič[3], Matic Kovačič[4], Uroš Zavrtanik[3], Sarah Haeserts[1, 2], Daniel Charlier[5], Janez Plavec[4], Alexander N. Volkov[1, 2, 6], Jurij Lah*[3], Remy Loris*[1, 2]

[1] *Structural Biology Brussels, Department of Biotechnology, Vrije Universiteit Brussel, Pleinlaan 2, 1050 Brussels, Belgium*

[2] *Centre for Structural Biology, VIB, Pleinlaan 2, 1050 Brussels, Belgium*

[3] *Department of Physical Chemistry, Faculty of Chemistry and Chemical Technology, University of Ljubljana, 1000 Ljubljana, Slovenia*

[4] *Slovenian NMR Center, National Institute of Chemistry, Hajdrihova, 19, 1000 Ljubljana, Slovenia*

[5] *Research group of Microbiology, Department of Biotechnology, Vrije Universiteit Brussel, Pleinlaan 2, 1050 Brussels, Belgium*

[6] *Jean Jeener NMR Centre, Vrije Universiteit Brussel, Pleinlaan 2, 1050 Brussels, Belgium*

*Corresponding authors: Jurij Lah, jurij.lah @fkkt.uni-lj.si and Remy Loris, remy.loris@vub.be

This document includes:

- Supplementary Tables 1 to 4
- Supplementary Figures 1 to 8

Supplementary Tables

Supplementary Table 1. Crystal data collection and refinement statistics.

	HigA2-Opr17
PDB code	8A0W
Diffraction source	SOLEIL synchrotron, Proxima I
Wavelength (Å)	0.97911
Temperature (K)	100
Detector	PILATUS 6M
Crystal-detector distance (mm)	509.7
Rotation range per image (°)	0.1
Total rotation range (°)	200
Space group	$P3_221$
a, b, c (Å)	94.0, 94.0, 123.7
α, β, γ (°)	90, 90, 120
Mosaicity (°)	0.092
Resolution range (Å)	49.26-2.33 (2.47-2.33)
Total no. of reflections	279349 (34557)
No. of unique reflections	27227 (2468)
Completeness (%)	99.2 (94.9)
Redundancy	10.3 (8.3)
$\langle I/\sigma(I) \rangle$	18.2 (2.0)
$R_{r.i.m.}$ / R_{merge}	0.078 (0.793)
CC1/2	99.9 (80.8)
Overall B factor from Wilson plot (Å^2)	54.1
R-work	0.174 (0.264)
R-free	0.199 (0.279)
Number of non-hydrogen atoms	1901
protein	1104
DNA	691
ligands	5
water	101
Protein residues	136

RMS (bonds, Å)	0.008
RMS (angles, °)	1.12
Ramachandran favored (%)	99.2
Ramachandran allowed (%)	0.8
Ramachandran outliers (%)	0
Clash score	2.12
Average B-factor (Å^2)	59.7
Protein atoms	53.4
DNA atoms	64.0
Ions	108
Solvent	63

Supplementary Table 2. Sequences of the proteins used in this study. HigA2 DNA binding C-terminal domain of HigA2 is underlined and the modified residues are shown in bold.

HigA2	SNRDLFAELSSALVEAKQHSEGKLTLKTHHVNDVGELNISPDEIVSIREQFNMSRGVFARLLHTSSRTLENWEQGRSVPNGQAVTLLKLVQRHPETLSHIAELHHHHHH
HigA2$_{\Delta IDR}$	ELNISPDEIVSIREQFNMSRGVFARLLHTSSRTLENWEQGRSVPNGQAVTLLKLVQRHPETLSHIAELHHHHHH
HigA2$_{IDR}$	NRDLFAELSSALVEAKQHSEGKLTLKTHHVNDVGELN**W**
HigA2$_{Shuff}$	**ERDGLTEFHSNLKLKLANVTSDVLSKAHVEHSQA**GELNISPDEIVSIREQFNMSRGVFARLLHTSSRTLENWEQGRSVPNGQAVTLLKLVQRHPETLSHIAELHHHHHH
HigA2$_{Phd}$	**ALDAEFASLFDTLDSTNKELVNRsgs**GELNISPDEIVSIREQFNMSRGVFARLLHTSSRTLENWEQGRSVPNGQAVTLLKLVQRHPETLSHIAELHHHHHH
HigA2$_{Mut(2-13)}$	**SSATASGATASS**LVEAKQHSEGKLTLKTHHVNDVGELNISPDEIVSIREQFNMSRGVFARLLHTSSRTLENWEQGRSVPNGQAVTLLKLVQRHPETLSHIAELHHHHHH
HigA2$_{Mut(14-25)}$	SNRDLFAELSSA**SSATASGATASS**TLKTHHVNDVGELNISPDEIVSIREQFNMSRGVFARLLHTSSRTLENWEQGRSVPNGQAVTLLKLVQRHPETLSHIAELHHHHHH
HigA2$_{Mut(26-37)}$	SNRDLFAELSSALVEAKQHSEGKL**SSATASGATASS**LNISPDEIVSIREQFNMSRGVFARLLHTSSRTLENWEQGRSVPNGQAVTLLKLVQRHPETLSHIAELHHHHHH

Supplementary Table 3. Oligonucleotide sequences used in this study.

Oligonucleotides used for amplifying DNA fragments for EMSA and footprinting.	
higBA2 oprF	GGGCGTTATAGCTCTTATCTG
higBA2 oprR	CCCAATATCTCGGTACTTTTCAAA
higBA2 interF	CAAAAATGAAATGTCTGACTTG
higBA2 interR	ATGACGCTGTACTAACTTTA
randF	CGACTCCTGCATTAGGAAGCAG
randR	CCTATATCGCCGACATCACCGATG
Oligonucleotides used for ITC, NMR and SAXS experiments. Inverted repeat is underlined.	
Opr25	ATCTGTACGCTTGGTGCGTACACTT
Opr33	TGCCATCTGTACGCTTGGTGCGTACACTTCCTA
Opr45	AATTCTTGCCATCTGTACGCTTGGTGCGTACACTTCCTATATGAA
Oligonucleotide used for crystallography.	
Opr17	GTACGCTTGGTGCGTAC
Oligonucleotides used sequencing pSB1A3 reporter vectors for the *in vivo* experiments.	
pSB1A3-F	TGCCACCTGACGTCTAAGAA
pSB1A3-R	ATTACCGCCTTTGAGTGAGC

Supplementary Table 4. SAXS data collection and analysis parameters.

	HigA2-Opr33	HigA2
Beamline	SWING	SWING
HPLC column		
Wavelength (Å)	1.03	1.03
q range	0.023 - 0.350	0.015-0.500
Temperature (K)	283	283
Exposure (ms/ms)	500/500	1000/500
I(0) / Guinier	0.0040	0.03
R_g (Å) /Guinier	26.2	23.5
I(0) / p(r)	0.0039	0.03
R_g (Å) / p(r)	26.5	23.3
R_g (Å) /model	27.0	23.9
V_C (Å^2)	342	250
V_C (Å^2) /model	356	238
D_{MAX} (Å)	90.7	93.7
Theoretical M_W (kDA)	42.1	22.7
Q_R (kDa)	39.7	21.6

Supplementary Figures

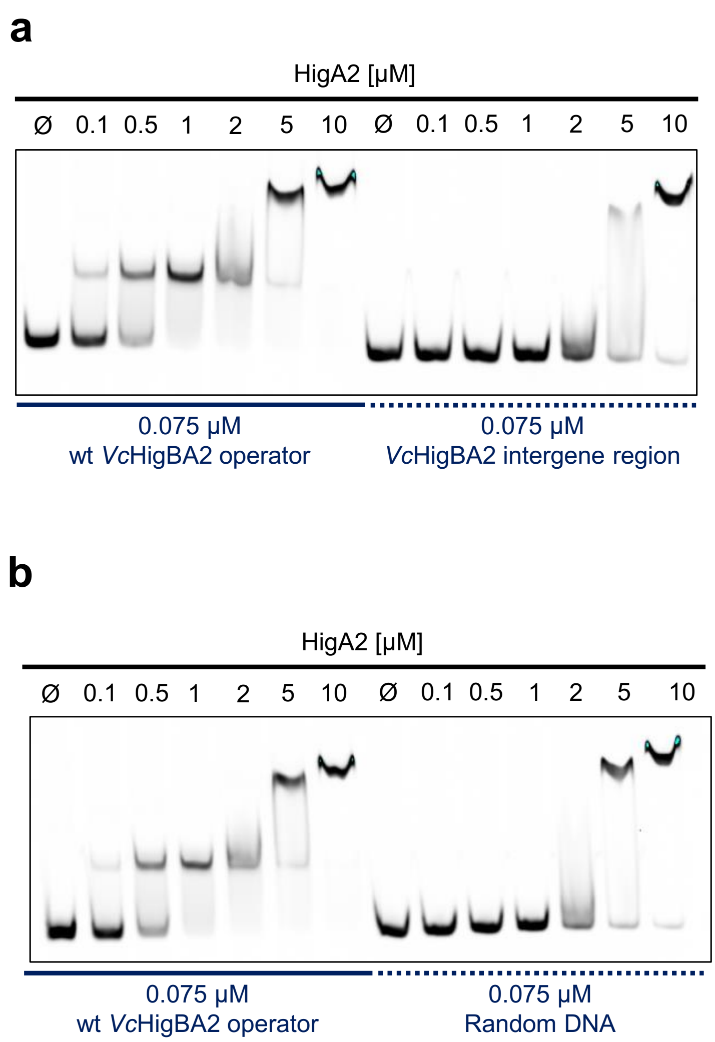

Supplementary Figure 1. HigA2 binds specifically to the *higBA2* operon. a EMSA of HigA2 antitoxin binding to the 140 bp DNA fragment containing *higBA2* operator region (amplified using primers *higBA2 opr*, Table S3) and to the 140 bp fragment containing *higBA2* intergenic region (amplified using primers *higBA2 inter*, Table S3), **b** and to the 140 bp fragment of unrelated DNA (primers *rand* in Table 3 amplify a 140 bp long region of the pet15b plasmid). The Ø lane corresponds to the free DNA, numbers indicate the µM concentrations of HigA2. DNA fragments were amplified using F primers (Table S3) with had Cy5 dye attached to the 5' end. Figure shows a representative data from one of the total n=2 independent experiments.

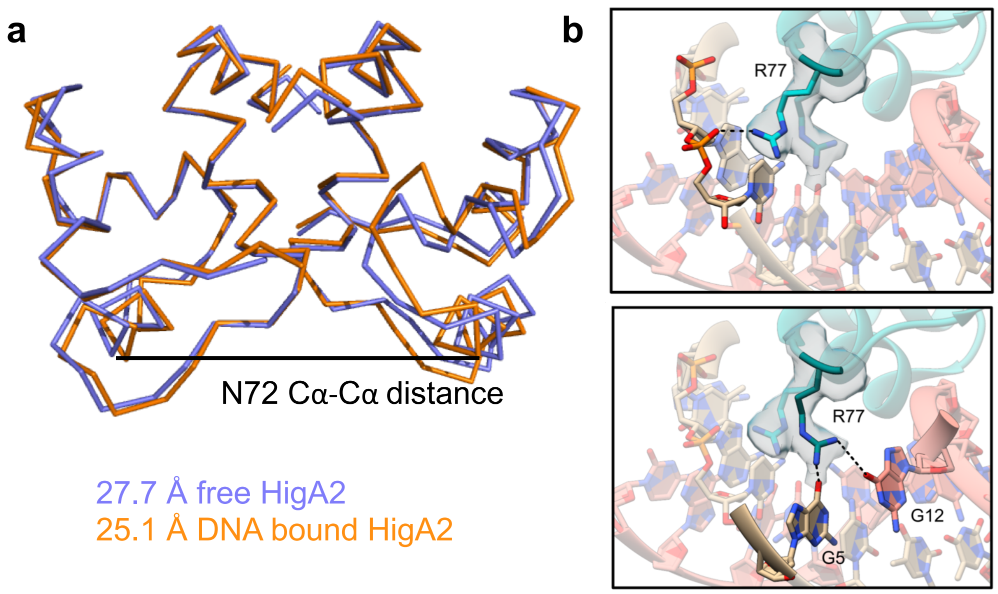

Supplementary Figure 2. Structural changes in HigA2. a Comparison of free and operator-bound HigA2. The backbone Cα atoms from the antitoxin C-terminal domain in its free state (PDB 5J9I) are shown in violet, while the operator-bound structure (PDB 8A0W) is shown in orange. The distance between recognition helix α3 is presented as a line, showing that the helices α3 move closer upon binding the operator. **b** In the crystal structure of HigA2 with Opr17 (PDB 5H9I) Arg77 adopts a double conformation and forms a hydrogen bond to the phosphate backbone of cytosine 4 (top) or a base-specific interactions to O6 of the guanine 5 on strand 1 and of the guanine 12 on strand 2 (bottom). The transparent surface shows Fo-Fc electron density contoured at 1 sigma around the Arg77 sidechain.

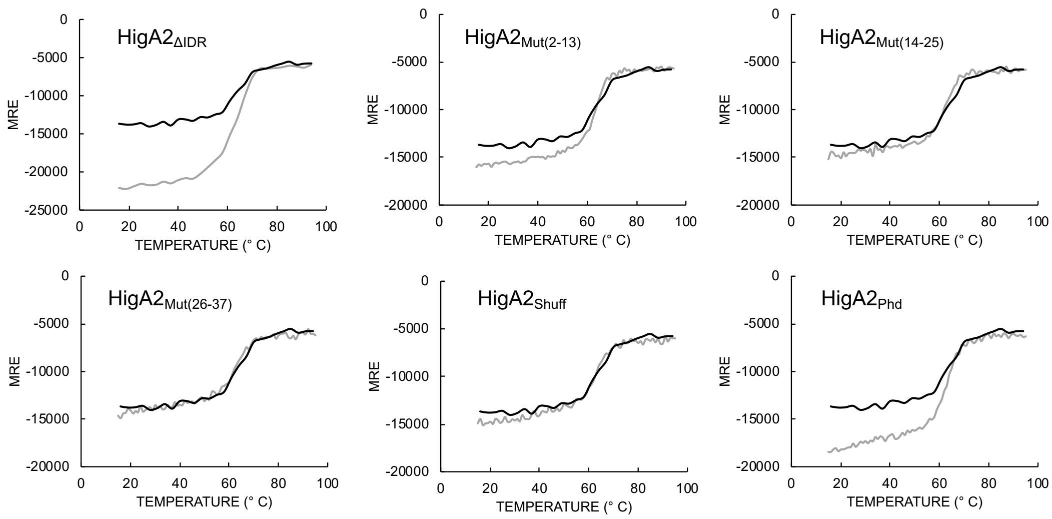

Supplementary Figure 3. Thermal stability of HigA2 and its variants. Mean residue ellipticity (MRE) at 222 nm as a function of temperature is shown for different HigA2 variants (grey) and wild-type protein (black). Stronger (more negative) intensity for some variants in low temperature region suggests that the construct is on average more helical, due to removal of the IDR (eg. HigA2$_{\Delta IDR}$) or disruption of the transient helix structure that is present in the wild-type HigA2.

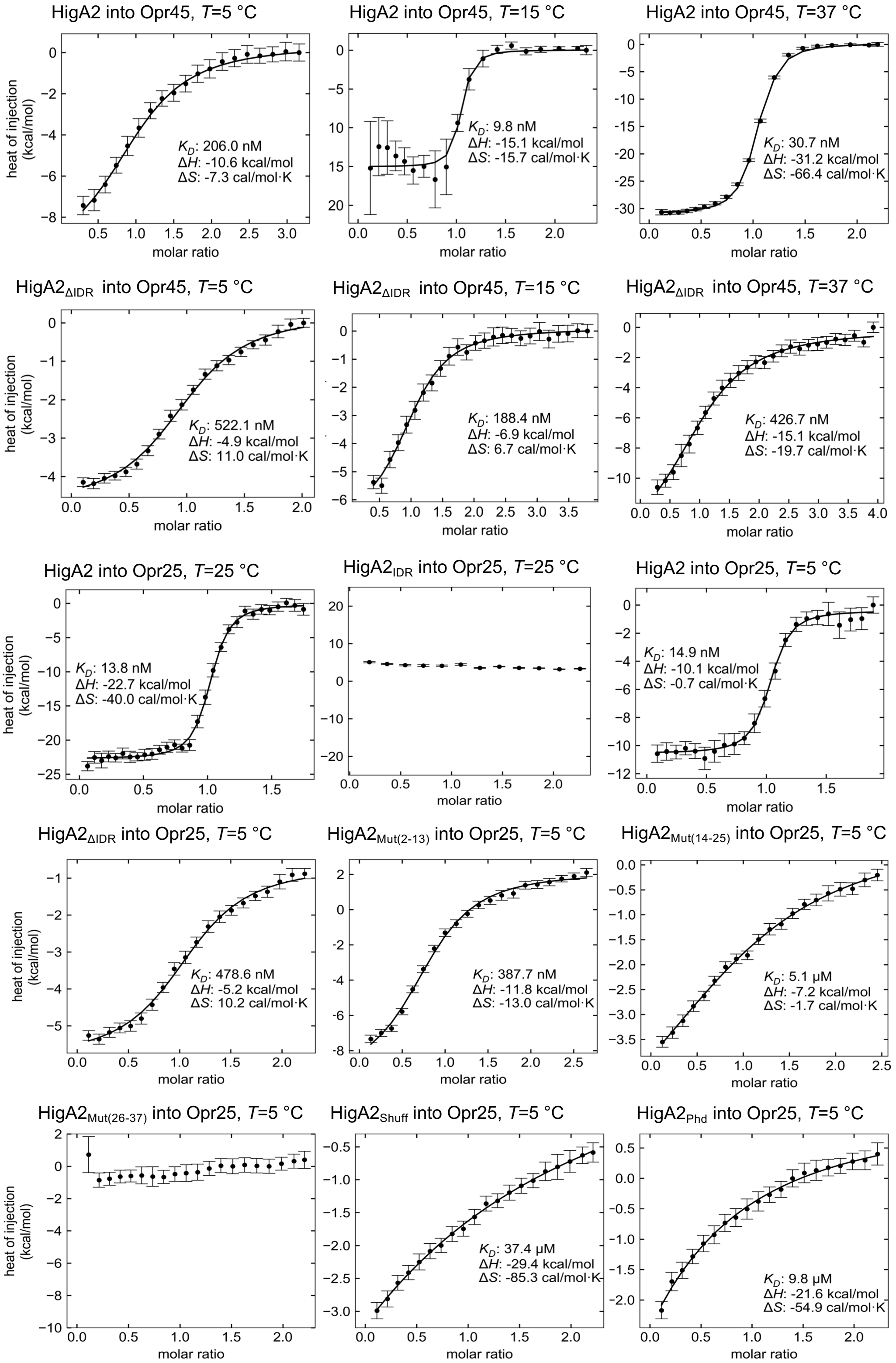

Supplementary Figure 4. HigA2-operator interactions monitored by ITC. Representative ITC isotherms show interactions of HigA2 or its variants with different operator fragments. Fits of the model function are shown as solid line, with the corresponding parameters listed in Table 1 and in each panel. Experiments for HigA2 and HigA2$_{\Delta IDR}$ binding to Opr45 at 25 °C are shown in the main text. Error bars indicate one s.d. of integrated heat effect.

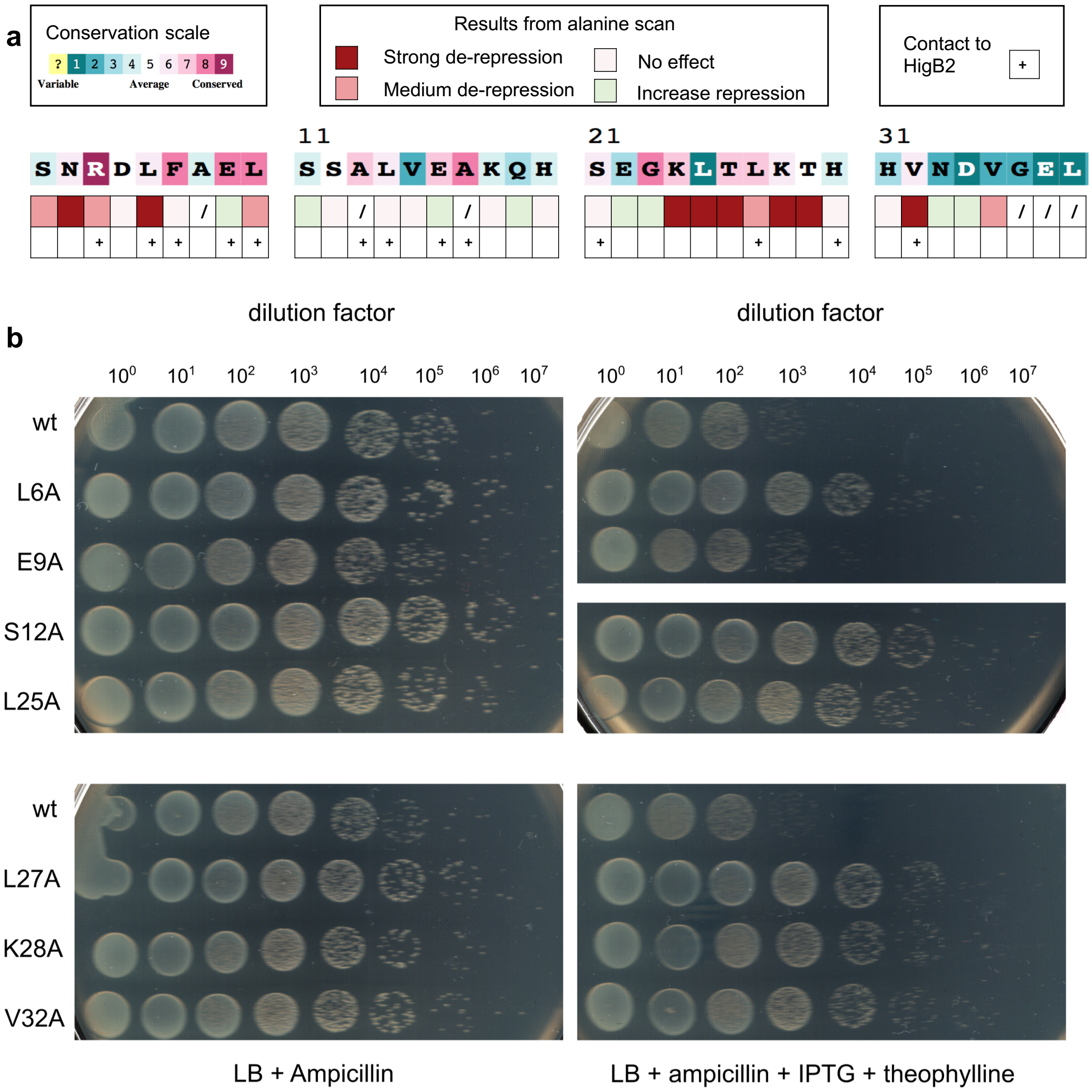

Supplementary Figure 5. Conservation of IDR residues and their functional roles for operator and toxin binding. a) Conservation of HigA2 IDR region is shown as color scale and was calculated with ConSurf using default parameters. Residues which bind HigB2 are labeled with plus sign, while those involved in operator interactions are color-coded (based on the alanine scanning results shown in Figure 3). **b**) Bacterial spotting assay shows that single alanine mutations in HigA2 IDR do not impair neutralization of the HigB2 toxin. Active toxin and different HigA2 mutants were placed under dual control vector with transcriptional control via Lac promoter and translational control via Theo riboswitch. Cells at different dilutions without inducers are shown on left, induced cells are shown on right. Interestingly for most alanine mutations viability of cells is even greater than for wt, which is perhaps related to improved stability (less degradation) of HigA2 mutants relative to wt. Importantly, none of these mutants decreases viability of cells. Figure shows a representative data from one of the total n=2 independent experiments.

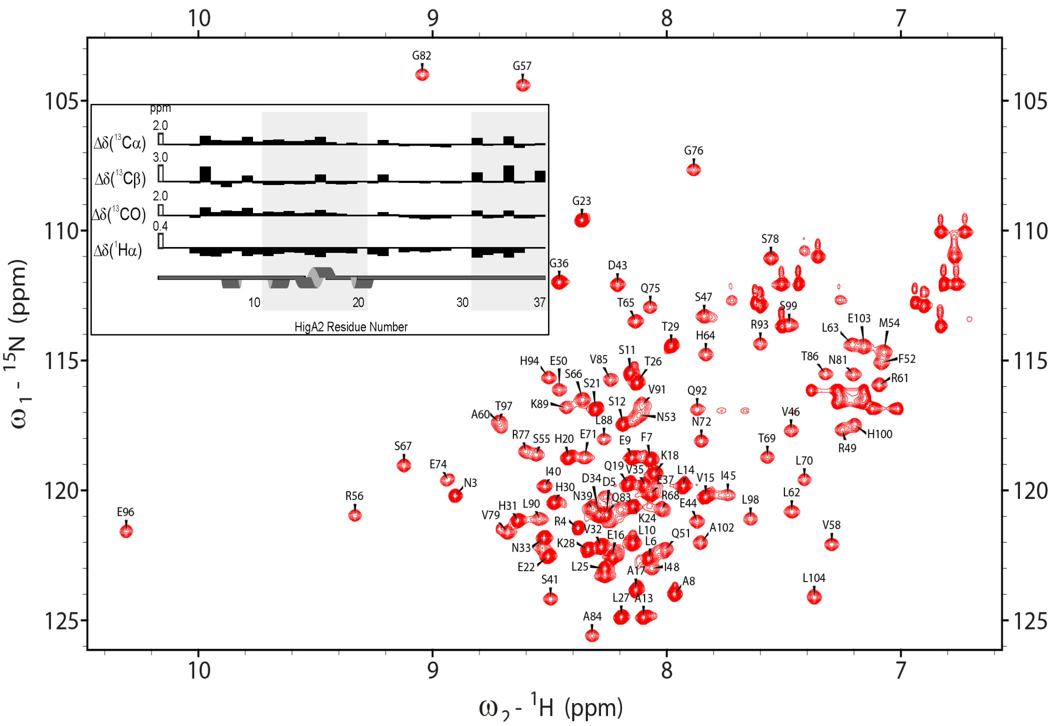

Supplementary Figure 6. Assignation of HigA2 backbone. a ^{15}N-HSQC spectrum of HigA2 homodimer protein with assigned backbone N-H cross-peaks. Conditions: 10/90 D_2O/H_2O solvent, 20 mM sodium phosphate buffer (pH 6.8), 130 mM NaCl, T = 25 °C, 800 MHz spectrometer. **Inset** Secondary structure of free HigA2 based on NMR chemical shift deviations. Threshold deviations from random-coil ^{13}Cα, ^{13}Cβ, ^{13}CO, and ^{1}Hα chemical shifts (Δδ) and the predicted secondary structure of the N-terminal part of the free HigA2. The open bars indicate the Δδ size for each nucleus type.

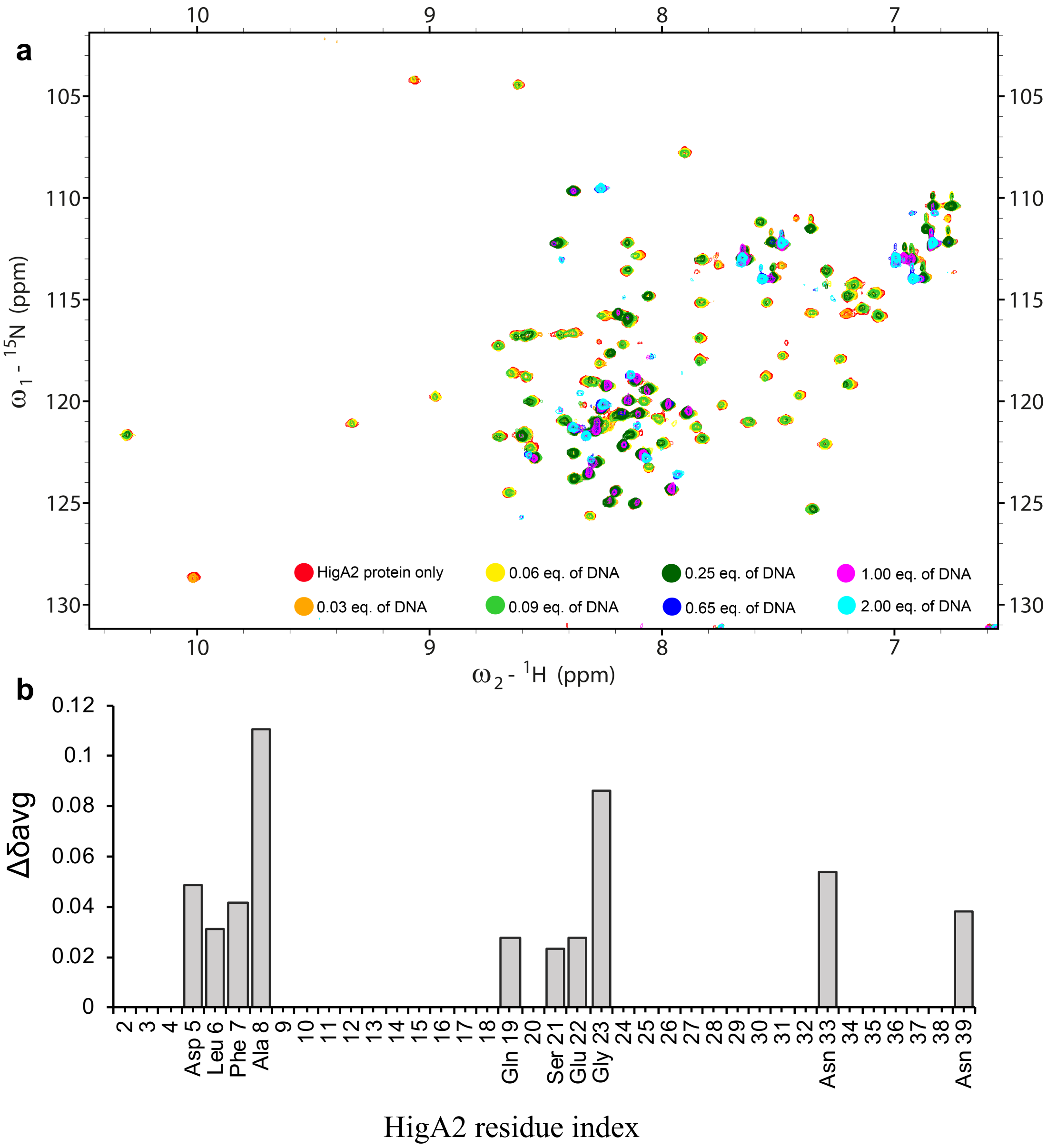

Supplementary Figure 7. Titration of operator DNA to HigA2. a) Overlay of eight 1H-15N HSQC spectra of HigA2 protein with various molar equivalents of 25 bp promoter DNA (Opr25). Titration was conducted on 37.5 μM HigA2 protein at pH 6.8 and 25 °C. Sample contained 20 mM sodium phosphate buffer and 130 mM NaCl. Spectra were recorded on 800 MHz NMR spectrometer. **b)** Histogram of the average chemical shift perturbations for backbone atoms between free HigA2 and HigA2 with 2.0 molar equivalents of Opr25 DNA. The resonances of these residues were explicitly assigned in the both free and bound HigA2.

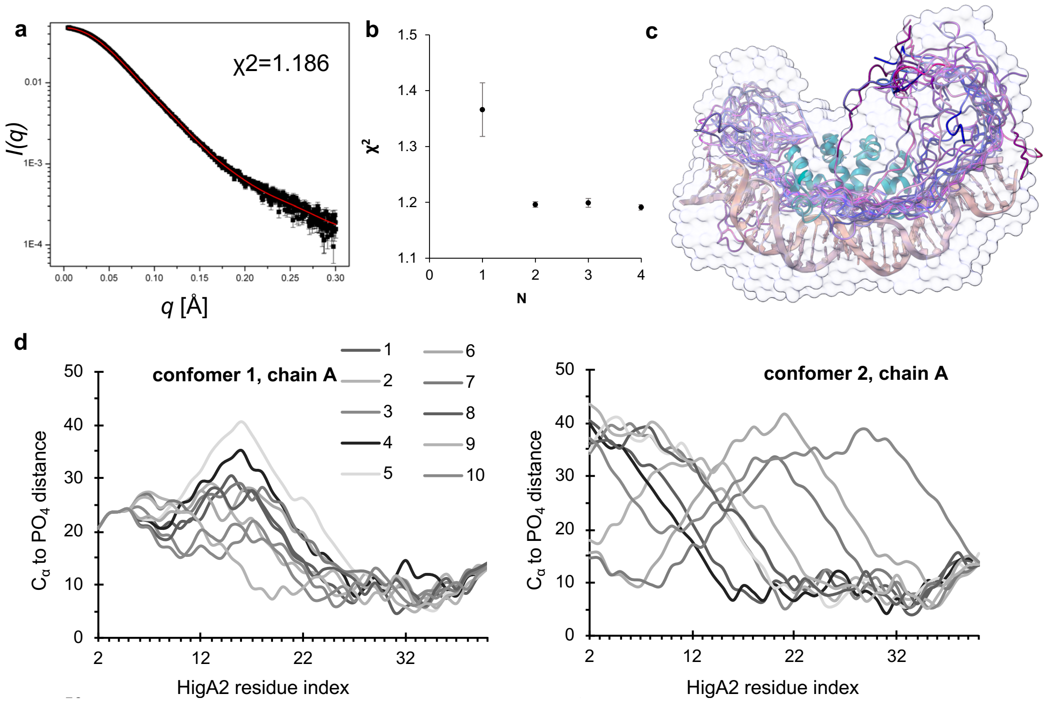

Supplementary Figure 8. HigA2-operator ensemble obtained by SAXS. a Comparison of the experimental scattering profile (black circles) and the profile corresponding to the HigA2-operator ensemble derived from the Xplor-NIH protocol (solid red line). **b** Quality metrics for calculation of HigA2-operator structural ensemble. Plot shows the variation of the SAXS $\chi2$ versus the ensemble size (N). **c** The 10-lowest energy structures of the HigA2-operator complex obtained from the Xplor-NIH protocol fit well into the molecular envelop calculated by DAMMIF. **d** Plots show distances (in Å) between the Cα atoms of HigA2 residues and the nearest phosphate atom of the DNA backbone for each of 10 structures of confomer 1 and 2.
