# Supplementary material for: Fuzzy recognition by the prokaryotic transcription factor HigA2 from Vibrio cholerae
Source: Nat Commun. 2024 Apr 10;15:3105. doi: 10.1038/s41467-024-47296-3 (PMC11006873; doi:10.1038/s41467-024-47296-3)
Supplement: Supplementary file 3 — Reporting Summary [file 41467_2024_47296_MOESM3_ESM.pdf]

nature portfolio

Corresponding author(s): Remy Loris, Jurij Lah

Last updated by author(s): 23-1-2024

Reporting Summary

Nature Portfolio wishes to improve the reproducibility of the work that we publish. This form provides structure for consistency and transparency in reporting. For further information on Nature Portfolio policies, see our Editorial Policies and the Editorial Policy Checklist.

Statistics

For all statistical analyses, confirm that the following items are present in the figure legend, table legend, main text, or Methods section.

n/a	Confirmed	
☐	☒	The exact sample size (n) for each experimental group/condition, given as a discrete number and unit of measurement
☒	☐	A statement on whether measurements were taken from distinct samples or whether the same sample was measured repeatedly
☒	☐	The statistical test(s) used AND whether they are one- or two-sided<br>*Only common tests should be described solely by name; describe more complex techniques in the Methods section.*
☒	☐	A description of all covariates tested
☒	☐	A description of any assumptions or corrections, such as tests of normality and adjustment for multiple comparisons
☐	☒	A full description of the statistical parameters including central tendency (e.g. means) or other basic estimates (e.g. regression coefficient) AND variation (e.g. standard deviation) or associated estimates of uncertainty (e.g. confidence intervals)
☒	☐	For null hypothesis testing, the test statistic (e.g. F, t, r) with confidence intervals, effect sizes, degrees of freedom and P value noted<br>*Give P values as exact values whenever suitable.*
☒	☐	For Bayesian analysis, information on the choice of priors and Markov chain Monte Carlo settings
☒	☐	For hierarchical and complex designs, identification of the appropriate level for tests and full reporting of outcomes
☒	☐	Estimates of effect sizes (e.g. Cohen's d, Pearson's r), indicating how they were calculated

Our web collection on statistics for biologists contains articles on many of the points above.

Software and code

Policy information about availability of computer code

Data collection	No custom code was used for data collection. ITC data were collected with the VPViewer 1.4.12 software (Malvern Panalytical, UK). CD spectra were recorded using JASCO Spectra Manager, while plate fluorescence was aquired using Tecan i-control software.
Data analysis	No custom code was used for data analysis. All other software is listed in main text and below:<br>Software used: PHENIX v1.12-2829-00, phenix.refine v1.12-2829-00, PHASER v1.12-2829-00, Coot v0.98, NITPIC 1.1.0, SEDPHAT 15.2b, NMRPipe 9.8, Cara 1.9.1, CCPNMR 2.5.2, ATSAS 3.0.2, Xplor-NIH v 2.49

For manuscripts utilizing custom algorithms or software that are central to the research but not yet described in published literature, software must be made available to editors and reviewers. We strongly encourage code deposition in a community repository (e.g. GitHub). See the Nature Portfolio guidelines for submitting code & software for further information.

Data

Policy information about availability of data

All manuscripts must include a data availability statement. This statement should provide the following information, where applicable:

- Accession codes, unique identifiers, or web links for publicly available datasets
- A description of any restrictions on data availability
- For clinical datasets or third party data, please ensure that the statement adheres to our policy

The crystallographic structural data for HigA2-Opr17 has been deposited into the Protein Data Bank under accession code 8A0W (https://www.rcsb.org/

structure/8A0W). The 1H, 13C and 15N chemical shifts for HigA2 have been deposited in the Biological Magnetic Resonance Data Bank under the Accession Number 51448 (https://bmrb.io/data_library/summary/index.php?bmrbId=51448). SAXS data has been deposited to SASDB (www.sasbdb.org) under codes SASDS76 (HigA2, https://www.sasbdb.org/data/SASDS76) and SASDS86 (HigA2-Opr25, https://www.sasbdb.org/data/SASDS86).

Research involving human participants, their data, or biological material

Policy information about studies with human participants or human data. See also policy information about sex, gender (identity/presentation), and sexual orientation and race, ethnicity and racism.

Reporting on sex and gender	N/A
Reporting on race, ethnicity, or other socially relevant groupings	N/A
Population characteristics	N/A
Recruitment	N/A
Ethics oversight	N/A

Note that full information on the approval of the study protocol must also be provided in the manuscript.

Field-specific reporting

Please select the one below that is the best fit for your research. If you are not sure, read the appropriate sections before making your selection.

- [x] Life sciences
- [ ] Behavioural & social sciences
- [ ] Ecological, evolutionary & environmental sciences

For a reference copy of the document with all sections, see nature.com/documents/nr-reporting-summary-flat.pdf

Life sciences study design

All studies must disclose on these points even when the disclosure is negative.

Sample size	No sample size calculation was performed given that tested hypotheses do not refer to any particular population. Sample size is stated in manuscript for each experiment separately.
Data exclusions	No data was excluded in the presentation. For the analysis of ITC thermograms the first point is omitted in all cases, as per standard practice related to the technical operation of the instrument. For the SAXS analysis truncated data was used (q < 0.3 Å-1) as per standard practice.
Replication	ITC experiments were performed with different batches of isolated proteins and repeated at least two times giving same results. In vivo repression experiments were performed in biological replicates (independent cell cultures) as stated in the main text either 3 or 6 times. EMSAs and bacterial spotting assays were performed twice giving same results.
Randomization	Not applicable, all experiment were performed in vitro
Blinding	Not applicable, all experiment were performed in vitro.

Reporting for specific materials, systems and methods

We require information from authors about some types of materials, experimental systems and methods used in many studies. Here, indicate whether each material, system or method listed is relevant to your study. If you are not sure if a list item applies to your research, read the appropriate section before selecting a response.

Materials & experimental systems

n/a	Involved in the study	
[x]	[ ]	Antibodies
[x]	[ ]	Eukaryotic cell lines
[x]	[ ]	Palaeontology and archaeology
[x]	[ ]	Animals and other organisms
[x]	[ ]	Clinical data
[x]	[ ]	Dual use research of concern
[x]	[ ]	Plants

Methods

n/a	Involved in the study	
[x]	[ ]	ChIP-seq
[x]	[ ]	Flow cytometry
[x]	[ ]	MRI-based neuroimaging

Plants

Seed stocks	N/A
Novel plant genotypes	N/A
Authentication	N/A
